# Nanostructures of Indium Gallium Nitride Crystals Grown on Carbon Nanotubes

**DOI:** 10.1038/srep16612

**Published:** 2015-11-16

**Authors:** Ji-Yeon Park, Keun Man Song, Yo-Sep Min, Chel-Jong Choi, Yoon Seok Kim, Sung-Nam Lee

**Affiliations:** 1 Department of Nano-Otpical Engineering, Korea Polytechnic University, Siheung, Gyeonggi 429-793 Republic of Korea; 2 Korea Advanced Nano Fab Center, Suwon, Gyeonggi 443-770 Republic of Korea; 3 Department of Chemical Engineering, Konkuk University, Seoul 143-701 Republic of Korea; 4 School of Semiconductor and Chemical Engineering, Semiconductor Physics Research Center, Chonbuk National University, Jeonju, Chonbuk 561-756 Republic of Korea; 5 Photonics Device Research Center, Korea Photonics Technology Institute (KOPTI), Gwangju 500-460 Republic of Korea

## Abstract

Nanostructure (NS) InGaN crystals were grown on carbon nanotubes (CNTs) using metalorganic chemical vapor deposition. The NS-InGaN crystals, grown on a ~5-μm-long CNT/Si template, were estimated to be ~100–270 nm in size. Transmission electron microscope examinations revealed that single-crystalline InGaN NSs were formed with different crystal facets. The observed green (~500 nm) cathodoluminescence (CL) emission was consistent with the surface image of the NS-InGaN crystallites, indicating excellent optical properties of the InGaN NSs on CNTs. Moreover, the CL spectrum of InGaN NSs showed a broad emission band from 490 to 600 nm. Based on these results, we believe that InGaN NSs grown on CNTs could aid in overcoming the green gap in LED technologies.

GaN-related compound semiconductors are prominent in the achievement of high-performance optoelectronic devices, such as light-emitting diodes, laser diodes, and field effect transistors, because they have wide direct band gaps of 0.6–6.2 eV^1^,^2^,^3^. While III-nitride thin films have been extensively developed for commercial optoelectronic devices with excellent physical, chemical, and synthesis properties, GaN-based nanostructures (NSs) are less developed in forms such as powders, quantum dots, nanorods, and nanowires^4^,^5^,^6^,^7^,^8^. Meanwhile, one-dimensional NSs of other materials have attracted significant attention for high-performance devices, because they provide good structure, properties, and applications^9^,^10^. The carbon nanotube (CNT) is a particularly important material because it may be applied in many technologies, including gas reservoirs, battery electrodes, and field-emission displays^11^,^12^. In the development of light-emitting diodes (LEDs), CNTs have been investigated as a current-spreading layer for improving light extraction efficiency, and for decreasing crystal defects in GaN film by the use of a CNT-patterned sapphire substrate^13^,^14^,^15^.

Since the commercialization of CNTs, much research has studied synthesis methods to achieve one-dimensional NS materials using CNTs as templates^16^,^17^,^18^,^19^,^20^. Among various nanoscale materials, carbide and nitride NSs, such as nanorods and nanowires, have been successfully grown on CNT templates^16^,^17^,^18^,^19^. With the fascinating properties of CNTs and III-nitrides, CNTs have also been used to synthesize GaN and InN nanorods and nanowires^11^,^19^,^21^,^22^,^23^,^24^,^25^,^26^. Most research groups have focused on binary III-nitrides, such as GaN or InN, to form nanotubes^21^,^26^, nanorods^22^, nanowires^19^, and nano-composites^23^,^24^,^25^ on CNTs. Ternary III-nitride systems, such as InGaN or AlGaN, have not been explored as much, because of the difficulty of growing highly uniform ternary NSs. To date, no reports have been made on the visible green emission of InGaN NSs grown on a CNT as a template. In this study, we focus on the growth and optical characterizations of InGaN NSs grown on CNT/Si templates.

## Results

### Metalorganic chemical vapor deposition growth of InGaN NSs on CNTs/Si template

**Figure 1:**
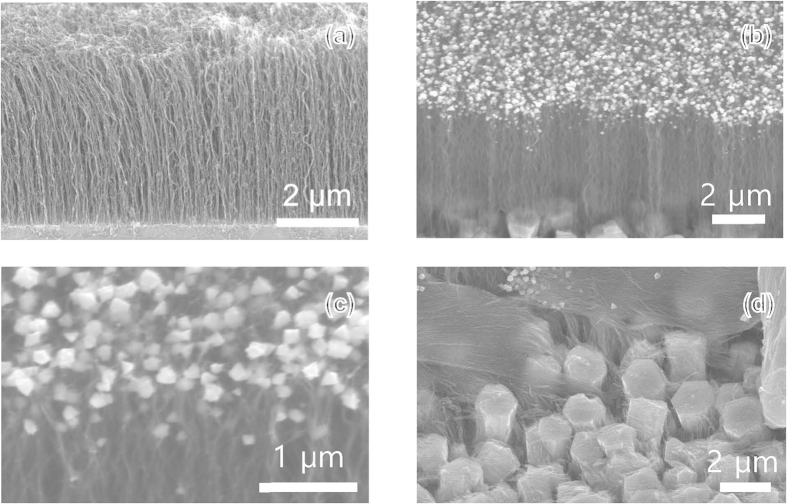
SEM images of InGaN NSs grown on CNT/Si templates. (**a**) SEM images of CNT forest, (**b**) InGaN NSs grown on CNT/SiO_2_/Si template using Fe/Al catalysts. (**c**) Highly magnified SEM image of InGaN NSs and (**d**) bottom images of CNT surrounded by GaN crystallites.

**Figure 2:**
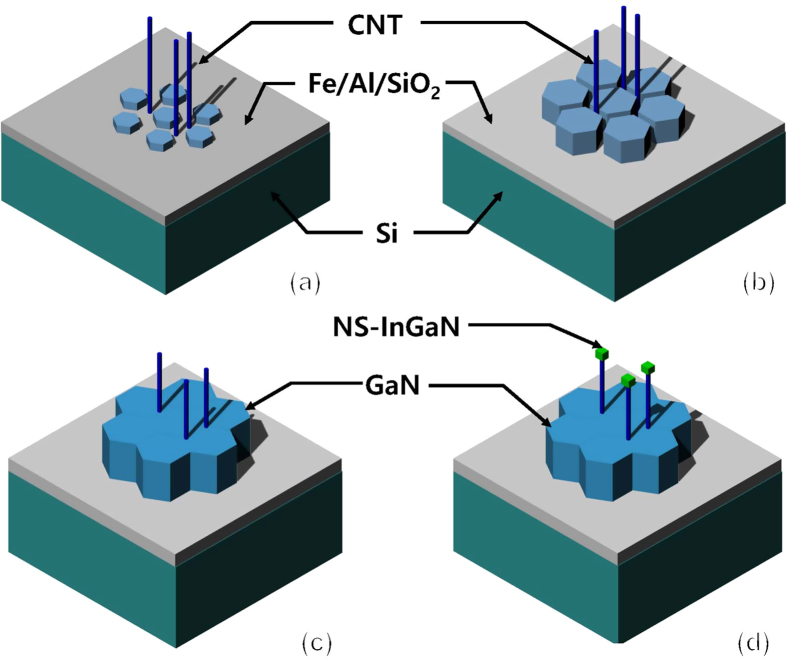
Schematic of growth mode ~ InGaN NSs on CNT template. Schematic growth mode ~ on CNT template. (**a**) The formation of GaN seed layer among CNTs, (**b**) the growth of GaN seed layer, (**c**) the CNT bundles encased by the coalescenced-GaN seed layer with large hexagon-shaped crystal, and (**d**) the growth of InGaN NSs on CNTs.

Figures 1(a,b) depict scanning electron microscopy (SEM) images of the CNT/Si template and the InGaN NSs grown on the CNT/Si template, respectively. Vertically aligned CNTs of 4.9 μm in length are observed on Fe/Al catalysts on the SiO_2_/Si template. The tilted SEM image shown in Fig. 1(b) shows the InGaN NSs grown vertically with respect to the substrate, indicating that InGaN NSs can be directly grown on a CNT/Si template using metalorganic chemical vapor deposition (MOCVD). The images indicate that InGaN NS formation was particularly successful at the ends of CNTs. The uniform coverage of InGaN NSs on the CNT bundles is shown in Fig. 1(c). From the SEM results, the InGaN NSs are polygonal crystal shapes with dimensions of 100–270 nm, which correspond to thermodynamically stable crystal planes, such as (0001) and (1010), in the wurtzite III-nitride crystal structure. The bundled CNTs are embedded with discontinuous hexagonal islands of crystals, which are not observed on the CNT/Si template shown in Fig. 1(a). SEM-energy dispersive X-ray spectroscopy (EDS) results show that these small hexagonal crystals are crystalline GaN, as shown in Fig. 1(b,d). Based on these results, we surmise that the nucleation and growth of hexagonal GaN crystals occurs on the regions of the SiO_2_/Si template with the Fe/Al catalysts where the Ga and N precursors pass through spaces between the CNTs, encasing the CNT bundles with hexagonal GaN crystals, as shown in Fig. 2(a–c).

### Crystallographic characterizations of InGaN NSs on CNTs/Si template

**Figure 3:**
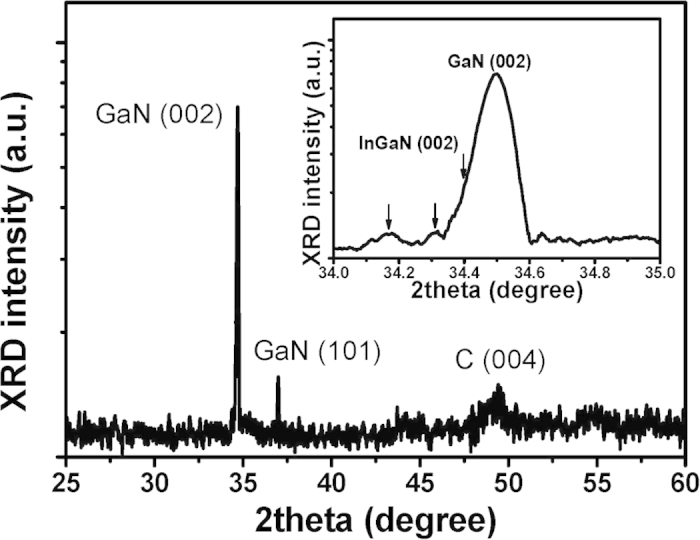
High-resolution X-ray diffraction (HR-XRD) Ω-2θ wide scans of InGaN NSs grown on CNT/Si templates. Two peaks are indexed to (001) GaN and (004) C. Inset: HR-XRD peaks of (002) InGaN and (002) GaN, indicating the nitride nanocrystals grown on CNT could be aligned to the (002) of the InGaN and GaN epilayer.

The structural and compositional properties of the InGaN NSs on CNT/Si template were characterized by high-resolution X-ray diffraction (HR-XRD). Figure 3 shows the high-resolution ω-2θ scan of InGaN NSs on CNT/Si template. Three peaks appear at 34.5°, 36.9°, and 49.8°. No peaks related to other components are observed at the resolution of the X-ray diffractometer. The peaks at 34.5° and 36.9° are indexed to the (002)_GaN_ and (101)_GaN_ planes, while the peak at 49.8° may be indexed to (004)_C_ originating from the CNT. This implies that the crystal planes of the GaN columns consist of (002)_GaN_ and (101)_GaN_. However, we believe that (002)_GaN_ was the main crystallographic plane, rather than (101)_GaN_, because the (002)_GaN_ peak is higher in intensity.

The inset of Fig. 3 shows the magnified (002)_GaN_ peak, which presents a very narrow full width at half maximum (FWHM) of 381.8 arc-seconds. This indicates that the GaN crystals mainly align with [0001]_GaN_, which is consistent with the hexagonal GaN columns observed in Fig. 1(d). Furthermore, the two low-angle side peaks and the asymmetric (002)_GaN_ peak, shown by the three arrow symbols in the inset, could be related to low In compositions of InGaN NSs. The XRD intensities of the two low-angle side peaks are very low, yet the peaks clarify the crystallinity of InGaN NSs, whereas the low-angle shoulder of the asymmetric (002)_GaN_ peak clearly represents the InGaN NSs with low In contents. From these results, despite the relatively low XRD intensity, the InGaN-related peaks located at low angles indicate that the In phases of the InGaN NSs may be separated into at least two components. We surmise that the In composition of the InGaN NSs may be different for each NS. The average grain size D of the InGaN NSs was estimated from the width of the diffraction peaks by the Debye-Scherer equation: D = (0.89λ)/(βcosθ), where β is the FWHM of the diffraction peak, θ is the angle of diffraction, and λ is the wavelength of the X-ray radiation^28^. The calculated average size of InGaN NSs is 89.9 nm, slightly smaller than that shown by the SEM results. The InGaN NSs dimension evaluated by XRD FWHM is the lower bound for the InGaN NSs, implying some strain in these grains.

### Structural characterizations of InGaN NSs on CNTs/Si template

**Figure 4:**
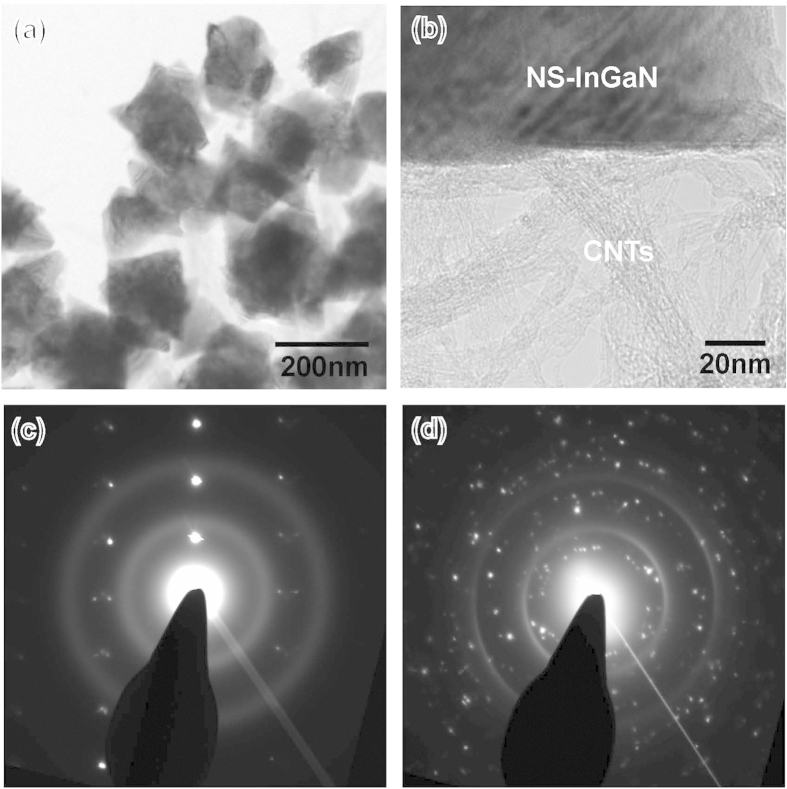
Crystallinity of InGaN NSs on CNT/Si template. (**a**) TEM images of CNT on Si substrate and (**b**) InGaN NSs. (**c,d**) TEM images of InGaN NSs on CNTs. The selected-area electron diffraction patterns (SADPs) of (**e**) multiple InGaN NSs and (**f**) one InGaN NS

The microstructural properties of InGaN NSs on the CNT/Si template were analyzed by transmission electron microscopy (TEM). Figure 4(a) shows that the InGaN NSs contain several types of polygonal crystals, which could be different thermodynamically stable facets. The dimensions of the InGaN NSs are evaluated to range from 100 to 200 nm, consistent with the SEM results (Fig. 1). Notably, the InGaN NSs are tightly bound to the CNTs, as shown in Fig. 4(b). The InGaN NSs were particularly well developed at the edges of the CNTs. This strongly suggests that InGaN NSs can be directly grown on CNTs. Furthermore, the selected area electron diffraction (SAED) pattern obtained from the interface between the InGaN NSs and the CNT shows well-defined sharp spots, corresponding to the^10^ plane of wurtzite InGaN, along with diffuse rings associated with the CNTs, as shown in Fig. 4(c). However, the SAED pattern obtained from several InGaN NSs crystallites exhibits numerous weak diffraction spots combined with sharp rings of diffracted intensity, as shown in Fig. 4(d), which is characteristic of a polycrystalline substance. Based on the SAED examinations, single-crystalline InGaN NSs were clearly grown on the CNT/Si templates, although the individual crystallites were oriented completely at random with respect to each other.

**Figure 5:**
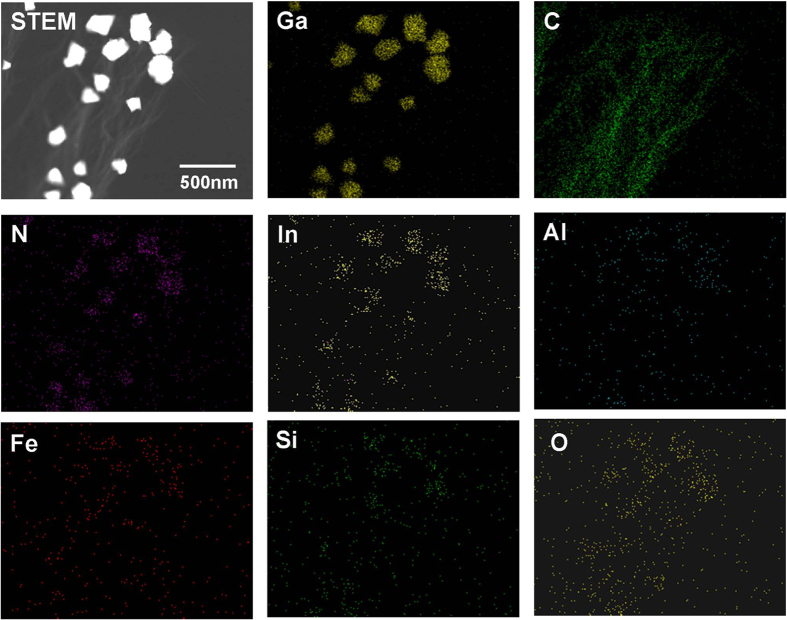
Compositional study of InGaN NSs grown on CNT/Si template using STEM. STEM image of InGaN NSs on carbon nanotube and STEM EDS mapping images of Ga, In, N, C, O, Si, Al, and Fe atoms.

Figure 5 presents the scanning transmission electron microscopy (STEM) Z-contrast image and corresponding EDS maps for Ga, In, and N atoms taken from the InGaN NSs grown on the CNT/Si template. The distributions of Ga, In, N and C atoms clearly match the positions of the InGaN NSs and CNTs shown in the STEM image. However, the distributions of Si, Al, and Fe atoms are inconsistent with the STEM image of the InGaN NSs on CNT within the detection limit of the instrument, indicating that the Si, Fe, and Al atoms do not diffuse from the Si substrate and Fe/Al catalyst with the growth of the InGaN NSs. From these results, we believe that InGaN NSs can be directly grown on CNTs without catalytic assistance.

### Excellent green emission from InGaN NSs on CNTs/Si templates

**Figure 6:**
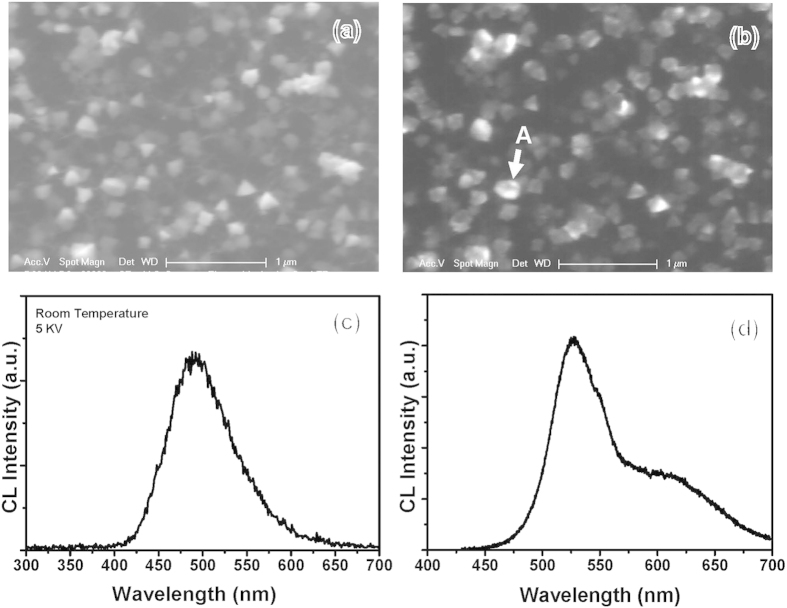
Optical properties of InGaN NSs grown on CNT/Si template. (**a**) SEM top image and (**b**) CL image of InGaN NSs/CNT. CL spectra of (**c**) one InGaN NSs and (**d**) multiple InGaN NSs.

Figure 6(a,b) depict SEM and the panchromatic cathodoluminescence (CL) images, respectively, of the InGaN NSs on the CNT/Si template. The bright CL image is strongly consistent with the SEM image of the InGaN NSs. This indicates the excellent optical properties of the InGaN NSs. We measured a particularly strong panchromatic CL emission from InGaN NSs on the CNTs, as shown in Fig. 6(c). The CL wavelength and FWHM of the CL spectrum are 493.4 and 79.4 nm, respectively. In general, the emission FWHM of the InGaN active layer of GaN-based LEDs is ~25 nm at ~500 nm, which is much lower than the FWHM of our InGaN NSs on CNTs. Despite growing the InGaN NSs on the CNT template, we surmise that this broad emission spectrum could be caused by the non-uniform In distribution or the broad size distribution of the InGaN NSs. To clarify the origin of the broad emission spectrum, we measured the CL spectrum of one InGaN NSs at position A, shown in Fig. 6(d). The spectrum shows two emission peaks at 528 nm and 612 nm. This implies that the broad emission of the InGaN NSs does not mainly originate from the size distribution of InGaN NSs, but from two or more In compositions among the InGaN NSs because InGaN NSs show a few crystallographic planes, yielding the different In incorporation rate to the different crystal planes.

## Discussion

**Figure 7:**
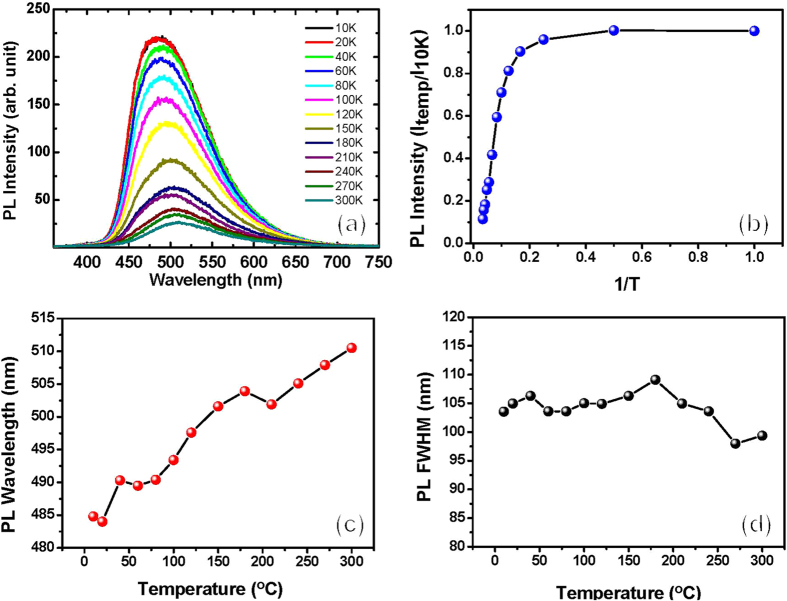
Temperature-dependent photoluminescence properties of InGaN NSs grown on CNT/Si template. (**a**) Temperature-dependent PL spectra of InGaN NSs/CNTs grown on Si from 10 to 300 K. (**b**) PL relative intensity, (**c**) emission wavelength, and (**d**) FWHM of InGaN NSs as functions of ambient temperature.

To further analyze the optical properties of the InGaN NSs, we performed temperature-dependent photoluminescence (PL) measurements from 10 K to 300 K, as shown in Fig. 7. Figure 7(a) shows the PL spectra at different ambient temperatures of the InGaN NSs grown on CNTs. The room-temperature PL emission wavelength and FWHM of InGaN NSs are 510.5 nm and 99.35 nm, respectively, slightly longer and broader than those measured by the CL spectra because of the different carrier excitation densities. However, we observe no GaN-related peak at 360 nm in the low-temperature PL spectra, indicating that the optical quality of GaN is much lower than that of the InGaN NSs. Our GaN crystal was grown at 800 °C, which is much lower than the >1000 °C growth temperature of high-quality GaN films, leading to the poor optical emission properties of the crystal. Therefore, we believe that our GaN crystal may assist the growth of high-quality InGaN NSs on CNTs as a seed layer. Based on Fig. 7(a), we have replotted the PL intensity ratios of the InGaN NSs as a function of reciprocal temperature, as shown in Fig. 7(b). The PL intensity ratio of 300 to 10 K is 11.4%, indicating the high internal quantum efficiency (IQE) of the InGaN NSs in the green emission region. Figure 7(c,d) show the temperature-dependent PL wavelengths and FWHMs as a function of ambient temperature. With decreasing the temperature from 300 to 10 K, the PL wavelength of InGaN NSs is shifted from 510.5 to 484.4 nm by the bandgap narrowing effect. However, the FWHMs of the PL spectra increase slightly with a temperature reduction from 300 to 180 K, and fluctuate at ~104 nm at temperatures below 180 K. The In incorporation in InGaN films is significantly affected by crystallographic plane orientation because they have different surface energies^29^. As shown in Fig. 4(a,d), the InGaN NSs grown on the CNT template contain several crystallographic planes. We surmise that the different facets of our InGaN NSs may have different In contents, generating the broad emission range from 10 to 300 K. Moreover, the low-temperature PL spectra of the InGaN NSs may induce other emission peaks around localized regions in the InGaN NSs with decreased ambient temperature, resulting in increased PL FWHMs at low temperatures.

In summary, we have demonstrated the successful growth of InGaN NSs on a CNT/Si template by MOCVD. SADPs indicated that the crystallites of InGaN NSs formed in random directions, and that only individual InGaN NSs were single crystals. From HR-XRD, the InGaN NSs crystallite was mainly aligned with the (0001) plane. A CL emission of ~500 nm was observed from the InGaN NSs crystallites. Temperature-dependent PL analyses indicated that the IQE of the InGaN NSs is 11.4% for the green emission region. Furthermore, the InGaN NSs exhibited temperature-independent PL FWHM behaviors from 10 to 300 K. This may result from the large localization of In in different facets of the InGaN NSs. We suggest that InGaN NSs on CNT/Si templates are among the best candidates for achieving green and yellow emission.

## Methods

### Growth of CNTs/Si template and InGaN NSs

Al was deposited in a 10-nm-thick film on a 200-nm-thick SiO_2_/Si (001) substrate by radio-frequency magnetron sputtering, and then oxidized at 650 °C in air to form the alumina supporting film. An ultrathin (~0.5 nm) Fe film was e-beam evaporated onto the alumina specimens and subsequently thermally oxidized at 600 °C for 10 min in air. After the e-beam evaporation process, the thickness of the catalyst film was measured by a thickness monitor using a quartz crystal microbalance. In order to grow the CNTs, the Fe/Al-deposited SiO_2_/Si (001) template was loaded into the reactor of a homemade radio-frequency (13.56 MHz) remote-plasma CVD^27^. As a source gas for the CNTs, methane gas was introduced at 60 sccm into the quartz tube reactor and the subsequent plasma (15 W) was ignited to grow the CNTs. During the growth of the CNT forest, the working temperature and pressure of the radio-frequency remote-plasma CVD were maintained at 450 °C and 64 Pa, respectively^27^. After growing the CNTs on the Fe/Al-deposited SiO_2_/Si (001) template, we loaded the template into the reactor of a MOCVD system to form the InGaN NSs on the CNT template. Trimethylgallium (TMGa), trimethylindium (TMIn), and ammonia (NH_3_) were used as precursors for Ga, In, and N, respectively. Before growing the InGaN NSs, we grew GaN on the CNT on Fe/Al-deposited SiO_2_/Si (001) template at 800 °C. The working pressure and V/III ratio were 27 kPa and 4300, respectively. Subsequently, InGaN NSs were grown by introducing TMGa, TMIn, and NH_3_ at 750 °C under a N_2_ atmosphere. As a result, we achieved InGaN NSs/CNTs grown on the Fe/Al-deposited SiO_2_/Si (001) template.

### Characterizations of InGaN NSs on CNTs/Si template

SEM and atomic force microscopy (AFM) were used to observe the surface structure of the InGaN NSs on the CNT/Si template. The optical properties of the InGaN NSs on CNT/Si template were characterized by CL analysis at room temperature using a Hitachi S-4700 system installed on a field-emission scanning electron microscope (FESEM). In addition, temperature-dependent PL spectroscopy was performed using a He-Cd laser (λ = 325 nm) with an excitation power density of 2.0 kW/cm^2^. The crystallinity of the InGaN NSs on CNT/Si template was characterized by HR-XRD and electron diffraction patterns. TEM examinations were performed with a Tecnai G2 F30 S-Twin (FEI) with an accelerating voltage of 300 kV and fitted with an EDS (EDAX Genesis) to characterize the atomic structure and the compositions of the InGaN NSs on the CNT/Si template.

## Additional Information

**How to cite this article**: Park, J.-Y. *et al.* Nanostructures of Indium Gallium Nitride Crystals Grown on Carbon Nanotubes. *Sci. Rep.* **5**, 16612; doi: 10.1038/srep16612 (2015).
